# Editorial: The Applications of New Multi-Locus GWAS Methodologies in the Genetic Dissection of Complex Traits

**DOI:** 10.3389/fpls.2019.00100

**Published:** 2019-02-11

**Authors:** Yuan-Ming Zhang, Zhenyu Jia, Jim M. Dunwell

**Affiliations:** ^1^Crop Information Center, College of Plant Science and Technology, Huazhong Agricultural University, Wuhan, China; ^2^Department of Botany and Plant Sciences, University of California, Riverside, Riverside, CA, United States; ^3^School of Agriculture, Policy and Development, University of Reading, Reading, United Kingdom

**Keywords:** genome-wide association study, mixed linear model, multi-locus model, mrMLM, omics big dataset

Since the establishment of the mixed linear model (MLM) method for genome-wide association studies (GWAS) by Zhang et al. (2005) and Yu et al. (2006), a series of new MLM-based methods have been proposed (Feng et al., 2016). These methods have been widely used in genetic dissection of complex and omics-related traits (Figure 1), especially in conjunction with the development of advanced genomic sequencing technologies. However, most existing methods are based on single marker association in genome-wide scans with population structure and polygenic background controls. To control false positive rate, Bonferroni correction for multiple tests is frequently adopted. This stringent correction results in the exclusion of important loci, especially for large experimental error inherent in field experiments of crop genetics. To address this issue, multi-locus GWAS methodologies have been recommended, i.e., mrMLM (Wang et al., 2016), ISIS EM-BLASSO (Tamba et al., 2017), pLARmEB (Zhang et al., 2017), FASTmrEMMA (Wen et al., 2018a), pKWmEB (Ren et al., 2018), and FASTmrMLM (Zhang and Tamba, 2018). Here we summarize their advantages and potential limitations for using these methods (Table 1).

**Figure 1:**
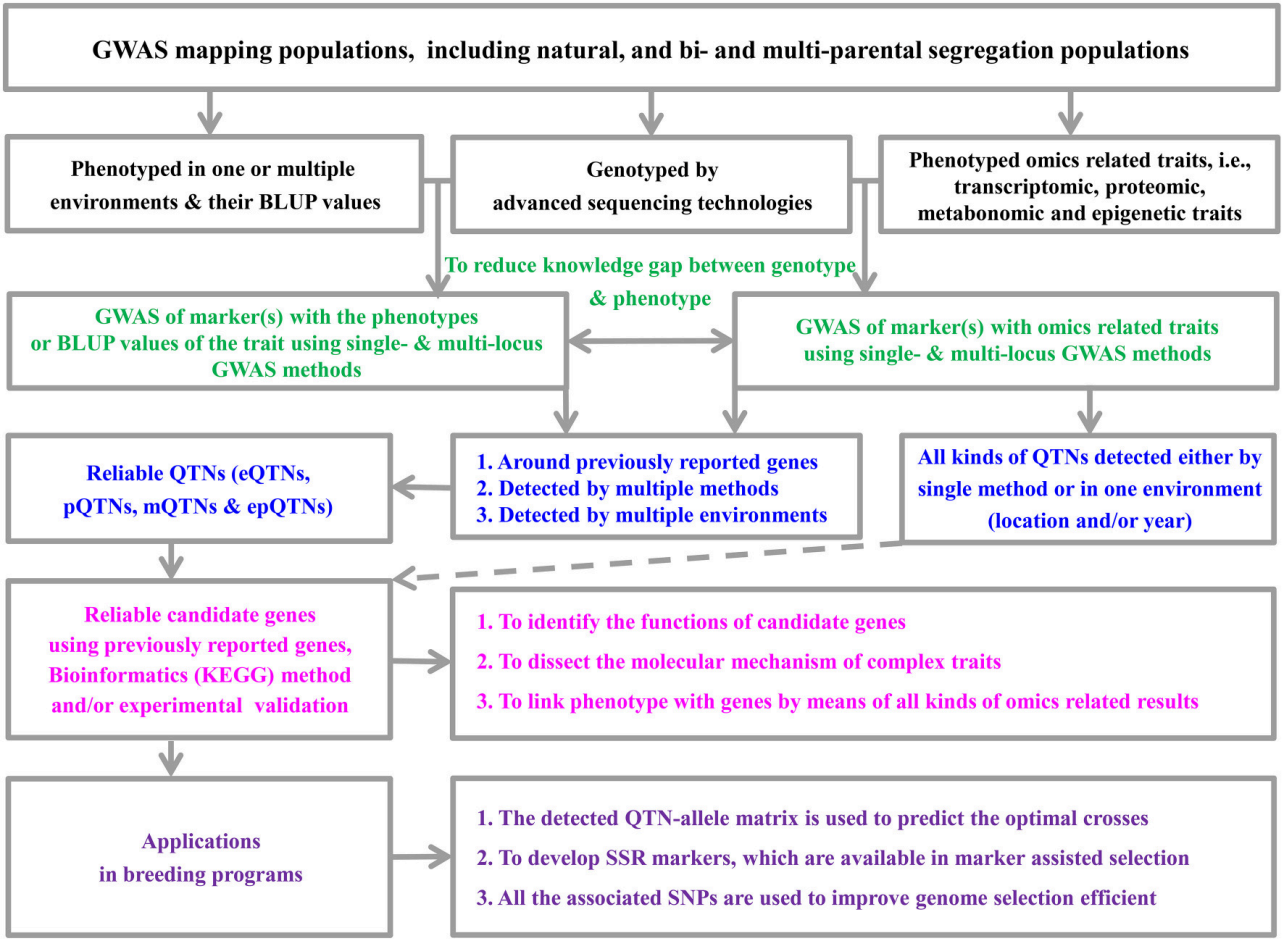
The pipeline framework of genome-wide association studies and their application.

**Table 1:** Comparison of single- and multi-locus GWAS methodologies.

	**Single-locus GWAS**	Multi-locus GWAS^*^
QTN detection power	Low	High
*P*-value threshold of significant QTN	5 × 10^−8^ (human genetics for common variants) 0.05/*m* ~ 1/*m* (crop genetics; *m* is no. of markers)	2 × 10^−4^ (or LOD = 3.0)
False positive rate	Low (with Bonferroni correction)	Low (with LOD = 3.0 or *P* = 2 × 10^−4^)
Multiple test correction	Yes	No
Polygenic background control	Yes	Yes (First step); No (Second step; all the potential genes have been included)
Population structure control	Yes	Yes
SNP effect	Fixed	Random
No. of variance components	Two (polygenic background and residual variances)	Three (QTN, polygenic background and residual variances; First step)
Multi-locus genetic model	No	Yes (second step)
How to reduce no. of variances	a) To fix the polygenic-to-residual variance ratio b) To estimate residual variance along with fixed effects	a) To fix the polygenic-to-residual variance ratio (1~5) b) To estimate residual variance along with fixed effects (1~4) c) Let the number of non-zero eigenvalues of ${\text{X}}_{\text{C}}{\text{X}}_{\text{C}}^{\text{T}}$ be one (3~5) d) To whiten the covariance matrix of polygenic K and noise (3~5)
Running time	Fast (GEMMA & EMMAX), slow (EMMA)	Fast (2, 6), slow (5), moderate (others)
Software	GEMMA: http://www.xzlab.org/software.html EMMAX: http://genetics.cs.ucla.edu/emmax	mrMLM: https://cran.r-project.org/web/packages/mrMLM/index.html mrMLM.GUI: https://cran.r-project.org/web/packages/mrMLM.GUI/index.html Parallel calculation with multiple CPU; quickly read big datasets; graphical user interface (GUI); To continuously run the programs for multiple traits

* *mrMLM, FASTmrMLM, FASTmrEMMA, pLARmEB, pKWmEB, and ISIS EM-BLASSO are marked by 1, 2, 3, 4, 5, and 6 respectively*.

## Multi-locus Genome-wide Association Studies for Complex Traits

### Comparison of GWAS Methodologies

Our methodological papers have showed their advantages in terms of quantitative trait nucleotide (QTN) detection power and QTN effect estimation accuracy over existing methods (Wang et al., 2016; Tamba et al., 2017; Zhang et al., 2017; Ren et al., 2018; Wen et al., 2018a). This conclusion has been echoed in a number of other applied studies in this Research Topic. For example, Ma et al. and Zhang et al. indicated that mrMLM, FASTmrEMMA, pLARmEB, and ISIS EM-BLASSO outperform the R package GAPIT, with ISIS EM-BLASSO being the most powerful multi-locus approach. Xu et al. compared one single-locus method (GEMMA) and three multi-locus methods (FASTmrEMMA, FarmCPU, and LASSO) in the genetic dissection of starch pasting properties in maize. As a result, FASTmrEMMA detected the most QTNs (29), followed by FarmCPU (19) and LASSO (12), and GEMMA detected the least QTNs (7). In the genetic dissection of salt tolerance traits in rice, Cui et al. compared all the six multi-locus approaches and identified the most co-detected QTNs from ISIS EM-BLASSO. Peng et al. used our six multi-locus GWAS methods to analyze 20 free amino acid levels in kernels of bread wheat (*Triticum aestivum* L.) and found the reliability and complementarity of these methods. In the detection of small-effect QTNs for fiber-quality related traits in the early-maturity varieties of upland cotton, Su et al. claimed that the multi-locus GWAS methods are more powerful and robust than the MLM method in TASSEL v5.0. Hou et al. demonstrated that 20 QTNs were associated with drought stress response using mrMLM, while three QTNs were associated with resistance to Verticillium wilt using EMMAX. Although the above studies have shown the advantages of multi-locus GWAS methods over single-locus GWAS methods, Chang et al., He et al., Li et al., and Xu et al. recommended the combination of single-locus methods and/or multi-locus methods to improve the detection power and robustness of GWAS, and Cui et al. recommended adding a bin analysis to the models or developing a hybrid method that merges the results from different methods. Our previous results in the analysis of real and simulated dataset support the above recommendations.

In addition, Liu et al. adopted four multi-locus GWAS algorithms (mrMLM, FASTmrEMMA, ISIS EM-BLASSO, and pLARmEB) to dissect the genetic foundation for fiber quality and yield component traits in RILs. As a result, a significant number of QTNs were found to coincide with the physical regions of the confidence intervals of reported QTLs, demonstrating the effectiveness and feasibility of multi-locus GWAS methods in RILs.

### The Critical *P*-Value or LOD Score for Significant QTN

In single-locus GWAS, one key concern is the high false positive rate (FPR). To reduce FPR, Bonferroni correction is frequently applied in the single-locus methods, including EMMAX (Kang et al., 2010), GEMMA (Zhou and Stephens, 2012), ECMLM (Li et al., 2014), and MLM (Yu et al., 2006). In human genetics, the genome-wide significance *P*-value threshold of 5 × 10^−8^ has become a standard for common-variant GWAS (Barsh et al., 2012; Fadista et al., 2016; Chang et al., 2018). However, this correction or the critical *P*-value in human genetics is too stringent to detect certain associated loci for complex traits in crop genetics. To address this issue, a modified Bonferroni correction has been proposed; in other words, the number of markers (*m*) in the correction formulas is replaced by the effective number of markers (*m*_*e*_) (Wang et al., 2016; Guan et al.). In real data analysis in crop genetics, some subjective and less stringent *P*-value thresholds for significant level are frequently applied owing to large experimental error, i.e., 1/*m* (*m* is the number of markers) (Li et al.; Xu et al.), 10^−5^ (Misra et al.), and 10^−4^ (Chang et al.). To balance high QTN detection power and low false positive rate, Xu et al. replaced Bonferroni correction by a less stringent criterion (1/*m*) for GEMMA, and a satisfactory result was achieved in their Monte Carlo simulation studies.

Theoretically, correction for multiple tests is unnecessary in multi-locus GWAS because all the potential genes or loci for complex traits are fitted to a single linear model and their effects are estimated and tested simultaneously. For example, 0.05 was chosen as the *P*-value threshold in QTN detection of Khan et al. (2018). Although relaxing the stringency of significance level in multi-locus GWAS can identify more hits, confidence in these hits will drop significantly. Thus, Segura et al. (2012) and Liu et al. (2016) imposed Bonferroni correction on QTN detection in their multi-locus GWAS methods. Our results indicated that Bonferroni correction in multi-locus GWAS of (Segura et al., 2012) and Liu et al. (2016) may be too stringent, while the cutoff of 0.05 in multi-locus GWAS of Khan et al. (2018) may be too relaxed due to the fact that a significance level of 0.05 can result in a high false positive rate. Lü et al. simply used LOD score ≥ 5 as a threshold for QTN detection in their multi-locus GWAS. Based on our studies, we proposed using LOD = 3.0 (or *P* = 0.0002) as a cutoff in multi-locus GWAS to balance the high power and low false positive rate for QTN detection.

### Heritability Missing in GWAS

Heritability missing is a common issue in GWAS (Maher, 2008). Human geneticists ascribe heritability missing to a few reasons, including rare alleles, gene-by-gene and gene-by-environment interactions, and miniature genetic effects of DNA variants that can hardly reach the level of genome-wide significance (Eichler et al., 2010). In our opinion, the stringent threshold in genome-wide detection is also a factor, because certain QTNs cannot meet the significant level if such *P*-value cutoff is applied. This viewpoint is supported by the simulation results of Xu et al.

In most GWAS methodologies, the genotypes of a SNP, for example, QQ, Qq, and qq, are conventionally coded as 2, 1, and 0, respectively. Thus, the estimated QTN effect is actually the average effect of allelic substitution, being *a* + (*q* − *p*)*d*. Let *a* + (*q* − *p*)*d* = 0, then *d* = *a*/(*p* − *q*). Where *p* takes different values, such as *p* = 0.1, 0.3, 0.5, 0.7, and 0.9, so *d* = −1.25*a*, −2.5 *a*, ∞, 2.5 *a*, and 1.25 *a*, respectively, indicating the difficulty in the detection of QTNs with over-dominance. This may be another reason for the heritability missing.

New methodologies have been proposed to handle heritability missing, for example, GCTA (Yang et al., 2011) and GREML-LDMS (Yang et al., 2015). In this Research Topic, we suggest that part of the missing heritability may be regained by using multi-locus GWAS methods, since more QTNs can be detected and overall estimated heritability will be increased.

## How to Determine Reliable QTNs and Mine Reliable Candidate Genes?

### How to Determine Reliable QTNs?

Firstly, when several multi-locus methods are used to analyze a same dataset, the QTNs identified by multiple approaches are usually reliable. For example, all the 31 genomic regions associated with four photosynthesis related traits were detected by at least three multi-locus methods in Lü et al., five QTNs associated with forage quality-related traits were detected by at least two methods in Li et al., and all the common QTNs either between single-locus methods and multi-locus methods, or across several multi-locus methods were declared in Misra et al. Secondly, the QTNs near previously reported trait-associated genes should be reliable. For example, the QTNs around genes *GRMZM2G163761, GRMZM2G412611*, and *GRMZM2G066749* likely contribute to the callus regenerative capacity (Ma et al.), the QTNs around genes *GRMZM2G032628* (*ae1*) and *GRMZM2G392988* may be associated with starch biosynthesis (Xu et al.), and the QTNs around genes *Gh_D102255* and *Gh_A13G0187* perhaps participate in cellular activities for fiber elongation (Liu et al.). Finally, the QTNs identified across various environments (locations and/or years) are also reliable, i.e., Liu et al. identified 57 QTNs that were associated with cotton fiber quality and yield components in at least two environments; Hu et al. repeatedly detected 39 QTNs clusters to be associated with 14 agronomic traits in 122 barley doubled haploid lines in multiple environments; Zhang et al. repeatedly detected 22 common QTNs to be associated with protein content in 144 soybean four-way recombinant inbred lines in 20 environments.

### How to Mine Reliable Candidate Genes?

All known genes in the regions around reliable QTNs potentially contribute to the traits of interest. However, only a subset of them may be reliable candidate genes which are worthy of further investigation. We can use homolog (previously reported genes) in other species, e.g., *Arabidopsis thaliana*, to mine reliable candidate genes in these regions. For example, *WOX2* in *Arabidopsis* has been reported to increase the rate of resistant seedlings from transformed immature embryos in maize and, therefore, the homologous gene *GRMZM2G108933* might play an important role in controlling maize callus regeneration (Ma et al.). Bioinformatics approaches, such as the KEGG pathway analytic tool, may be used for mining reliable candidate genes and relevant gene networks. For example, two genes (*LOC_Os01g45760* and *LOC_Os10g04860*) are found to be involved in auxin biosynthesis in rice using KEGG (Cui et al.). Experimental validations are often needed to confirm the associations between these candidate genes and the traits of interest. For instance, RNA-seq analysis and qRT-PCR experiments verified that four genes (*RD2, HAT22, PIP2*, and *PP2C*) are associated with drought tolerance in cotton (Hou et al.); genomic DNA sequencing showed that two candidate genes *BnaA08g08280D* and *BnaC03g60080D* are different between the high- and low-oleic acid lines (Guan et al.). The combined use of GWAS and experimental validation has great potential for detection of new genes and their biological functions. For example, a new gene *GRMZM2G065083* was found by Xu et al. to play a critical role in starch biosynthesis in maize by being involved in the gluconeogenesis process, hexose biosynthetic and metabolic process, and glucose-6-phosphate isomerase activity, providing insights into the molecular mechanism underlying the pasting properties of maize starch.

Important genes may be missed if we only select consensus QTNs identified by more than one methodology or in more than one experiment/environment. In practice, we found that some QTNs detected by only one multi-locus method or one environment may lead to important discoveries. These QTNs may be used to mine candidate genes through network analysis using bioinformatics analysis and/or experimental validation.

### How to Make Use of the GWAS Results?

The main product of GWAS includes the detected QTNs and the candidate genes nearby. Three approaches are available for applying these results to breeding programs. Firstly, one can organize the detected QTN-allele matrix as the population genetic constitution to facilitate the selection of optimal crosses. For example, the top 10 optimal crosses were predicted according to their 95th percentile weighted average values (Khan et al., 2018). Secondly, we can develop SSR markers around the reliable QTNs and utilize them in marker assisted selection of crops (Li et al., 2018). Thirdly, all the SNPs that are significantly associated with the trait of interest can be used for improving genome selection (He et al.; He et al., 2019).

Figure 1 summarizes how to design a GWAS to identify QTNs and mine candidate genes, of which the biological functions may be further investigated or validated at a molecular level.

## Future Perspectives

It is becoming common to use multiple statistical methods to detect major quantitative trait loci (QTLs) in the linkage analyses of complex traits. Thus, we recommend using a few GWAS methods, especially several multi-locus GWAS methods which do not need correction for multiple comparisons, to investigate complex traits. However, not all QTNs can be identified by all these methods, posing difficulties for using these GWAS results. This may be ascribed to the fact that the various GWAS models are based upon different genetic or statistical assumptions. Possible solutions have been provided in this editorial to compare the results from various GWAS models and screen for candidate QTNs or genes, facilitating the subsequent validation or application.

Interaction at different omics levels, including QTL-by-environment and QTL-by-QTL interactions, can be detected with various software programs in linkage analysis. Nevertheless, methods and software programs with comparable function are quite limited in GWAS, especially for the studies of quantitative traits in natural populations where large numbers of genomic markers are analyzed. The number of variables in GWAS models will increase sharply if interactions are considered, challenging both computational efficiency and detection power. Multicollinearity among highly saturated and linked markers is another issue in GWAS, which impairs the efficiency and accuracy of the current statistical methods. Innovative strategies are needed to distill many thousands of variables by removing the redundant genomic markers such that the computational burden and impact from multicollinearity can be reduced and the studies of interactions made more feasible.

Zhang et al. (2018) showed that the explained heritability increases with sample size in GWAS, and also estimated that the required sample size may range from a few hundred thousand to multiple millions to account for most of the heritability. The samples used in crop genetics, however, is often small, therefore, increasing sample size in crop GWAS has a great potential in future research.

With the rapid advances in various technologies, other types of omic data, including transcriptomic, proteomic, metabolomic and epigenetic data, have been recently exploited in crop research (Peng et al.; Wen et al., 2018b). These multi-omic variables may be treated as additional traits in GWAS, which promises to reduce knowledge gap between genotype and phenotype and will eventually benefit selective breeding. For example, omic-traits (at various layers) that are mapped to the same genomic locations with agronomic traits will provide multi-dimensional insights of genetic architectures and the underlying biological pathways. We believe multi-locus GWAS methodologies will become useful and popular tools for analysis of omics big datasets and help understand the mysterious world of genetics.

## Author Contributions

Y-MZ, ZJ, and JMD contributed to manuscript writing, provided important interpretations, and revised the work. All the authors checked and confirmed the final version of the manuscript.

### Conflict of Interest Statement

The authors declare that the research was conducted in the absence of any commercial or financial relationships that could be construed as a potential conflict of interest.
